# Supplementary figures and images for: Next Generation Sequencing-Based Transcriptome Predicts Bevacizumab Efficacy in Combination with Temozolomide in Glioblastoma
Source: Molecules. 2019 Aug 22;24(17):3046. doi: 10.3390/molecules24173046 (PMC6749405; doi:10.3390/molecules24173046)

## Slide 1
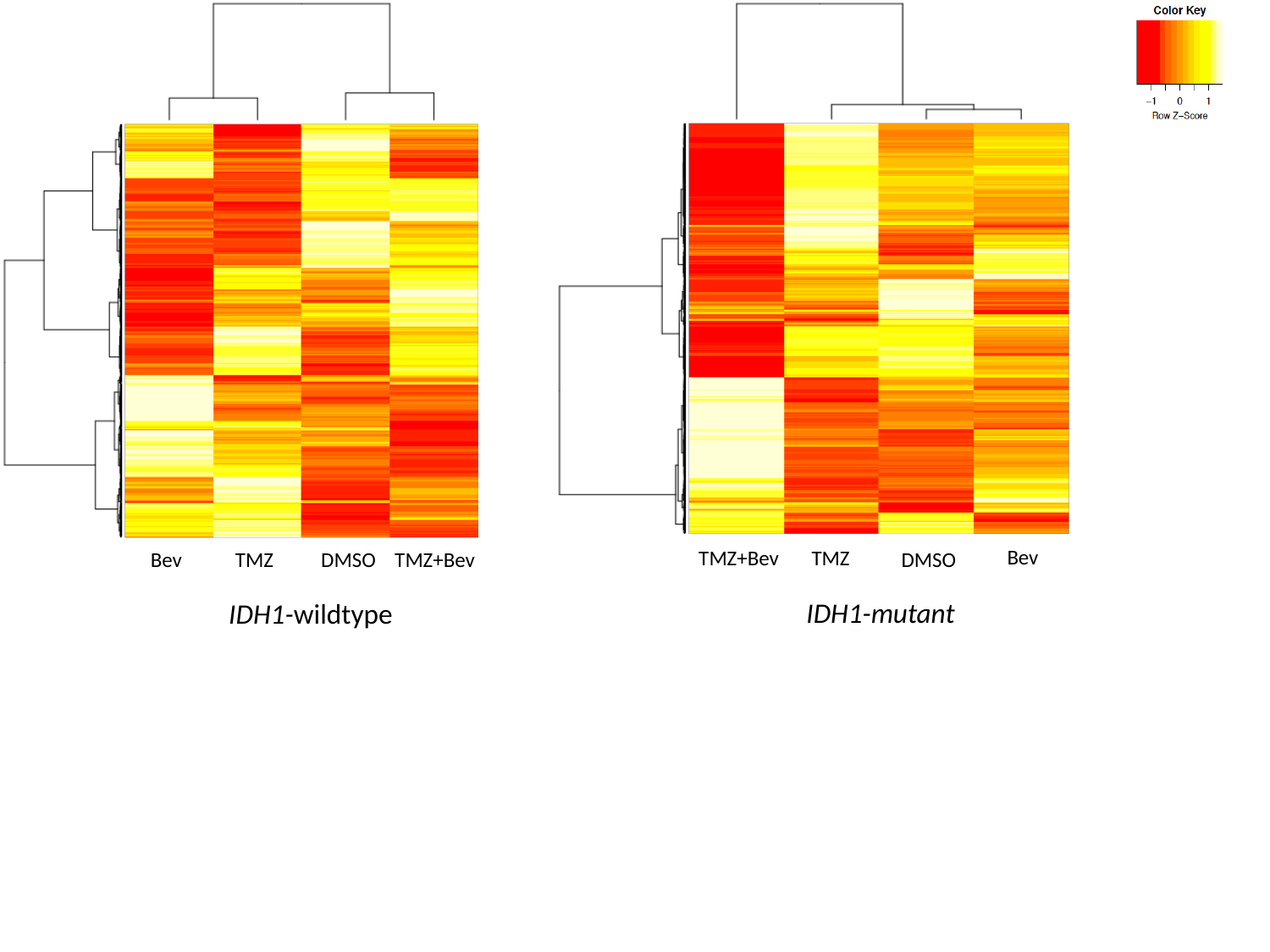

Bev
TMZ
TMZ+Bev
DMSO
TMZ
DMSO
TMZ+Bev
Bev
IDH1-mutant
IDH1-wildtype

Supplement: Supplementary file 1 [file molecules-24-03046-s001.zip › molecules-557251-SI.pptx]
